# Development and Comparison of Conventional and 3D-Printed Laboratory Models of Maxillary Defects

**DOI:** 10.3390/dj11050115

**Published:** 2023-04-27

**Authors:** Ahmad Alanezi, May Aljanahi, Keyvan Moharamzadeh, Ahmed Ghoneima, Abdel Rahman Tawfik, Amar Hassan Khamis, Moosa Abuzayeda

**Affiliations:** 1 Hamdan Bin Mohammed College of Dental Medicine (HBMCDM), Mohammed Bin Rashid University of Medicine and Health Sciences (MBRU), Dubai P.O. Box 505055, United Arab Emirates; 2 Dubai Dental Hospital (DDH), Dubai P.O. Box 505097, United Arab Emirates; 3 School of Clinical Dentistry, University of Sheffield, Sheffield S10 2TA, UK

**Keywords:** maxillary defect, impression, 3D printing, digital dentistry, prosthodontics

## Abstract

Background: Recording accurate impressions from maxillary defects is a critical and challenging stage in the prosthetic rehabilitation of patients following maxillectomy surgery. The aim of this study was to develop and optimize conventional and 3D-printed laboratory models of maxillary defects and to compare conventional and digital impression techniques using these models. Methods: Six different types of maxillary defect models were fabricated. A central palatal defect model was used to compare conventional silicon impressions with digital intra-oral scanning in terms of dimensional accuracy and total time taken to record the defect and produce a laboratory analogue. Results: Digital workflow produced different results than the conventional technique in terms of defect size measurements which were statistically significant (*p* < 0.05). The time taken to record the arch and the defect using an intra-oral scanner was significantly less compared with the traditional impression method. However, there was no statistically significant difference between the two techniques in terms of the total time taken to fabricate a maxillary central defect model (*p* > 0.05). Conclusions: The laboratory models of different maxillary defects developed in this study have the potential to be used to compare conventional and digital workflow in prosthetic treatment procedures.

## 1. Introduction

Rehabilitation of maxillectomy defects can be challenging and often requires a multidisciplinary team approach to treatment [1,2]. Maxillary defects can be caused by surgical treatment of malignant lesions, congenital malformations, and trauma. Communication between the oral and nasal cavities can affect oral function, including disorders in phonation and the inability to chew and swallow [3]. Patients can also be affected psychologically, due to the impact on both quality of life and aesthetics [4]. Such defects may be restored with tissue grafting, surgical reconstruction, or obturator prostheses depending on the condition of the patient [5]. Knowing how to record accurate impressions of maxillectomy defects is, therefore, very crucial for prosthetic treatment, reducing chair-side time, and providing the patient with greater confidence. 

Maxillectomy deformities have been categorized by Brown and Shah as either horizontal or vertical midface defects [6]. Vertical classification includes (I) maxillectomy not causing an oronasal fistula; (II) not involving the orbit; (III) involving the orbital adnexae with orbital retention; (IV) with orbital enucleation or exenteration; (V) orbitomaxillary defect; and (VI) nasomaxillary defect. Horizontal classification includes (a) palatal defect only, not involving the dental alveolus; (b) less than or equal to 1/2 unilateral; (c) less than or equal to 1/2 bilateral or transverse anterior; and (d) greater than 1/2 maxillectomy.

**Figure 1:**
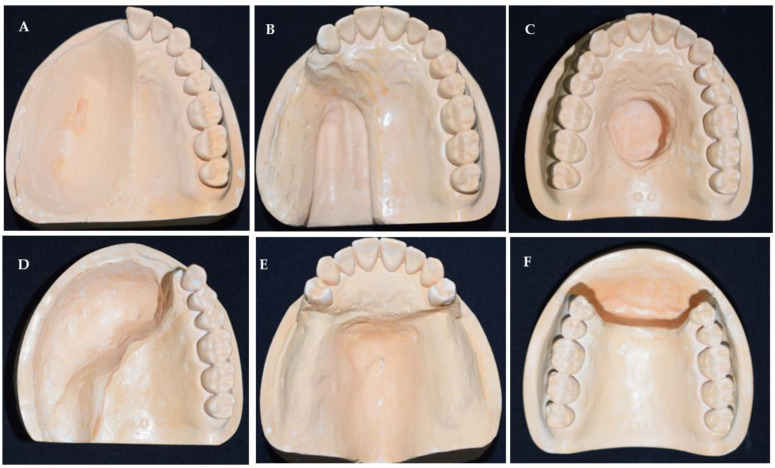
Stone models of six types of maxillary defects based on Aramany’s classification showing (**A**) midline resection, (**B**) unilateral resection, (**C**) central palate resection, (**D**) bilateral anteroposterior resection, (**E**) posterior defect, and (**F**) anterior defect.

One of the widely used classification systems for maxillectomy defects in the past was designed by Aramany in 1978 and includes six groups [7] as described below based on partially edentulous maxillary dental arches (Figure 1):

- Class I: Midline resection
- Class II: Unilateral resection
- Class III: Central palate resection
- Class IV: Bilateral anteroposterior resection
- Class V: Posterior resection
- Class VI: Anterior resection

Prosthetic reconstruction is often favoured, not only due to ease of treatment and cost-effectiveness, but also because a prosthesis prevents mucus accumulation and nasal infections and means that the underlying tissue can be monitored for tumour recurrence [8]. 

Prosthetic reconstruction is usually performed by an obturator; however, they are challenging to fabricate as different defect configurations require specific considerations. According to the Glossary of Prosthodontics Terms, an obturator is a prosthesis used to close a congenital or an acquired tissue opening, primarily of the hard palate and/or contiguous alveolar structures [9].

Obturators are used throughout treatment and can be classified into surgical, interim, and definitive. 

Good obturators contribute to a better quality of life and may improve the psychological status of the patient [10]. On the other hand, impaired obturator functioning and handling can do more harm than good, leading to deficits in speech, mastication, swallowing or facial disfigurement thereby resulting in patient dissatisfaction [11,12].

For many years, prosthodontists have struggled to acquire the necessary level of accuracy and safety in making impressions for these patients, and with a shift in the direction of digital dentistry, it may be beneficial to explore these options in maxillectomy patients. This can increase the ease with which impressions are taken, shorten the chairside time, and ultimately provide a solution for clinicians with less experience in taking impressions using the conventional approach for these patients. 

With the help of digital dentistry, the complex process of fabricating a prosthesis can be simplified, shortening the time acquired. The development of a digital impression reduces the number of times a patient must endure invasive procedures. Additionally, 3D printing is swiftly developing in healthcare and across all dental specialties. However, despite significant progress, there is a paucity of knowledge on how specialists employ additive technology and will require training [13]. The advantages of using 3D printing and technology provides health and safety advantages, as the presence of an oral–nasal communication can increase the risk of a patient aspirating impression material which can lead to serious problems [14,15]. Generally, the conventional impression-making process poses a risk to maxillectomy patients and their prosthodontists, so it is not surprising that digital acquisition and digital impressions are being utilised in fixed prosthodontics with clinicians increasingly using such techniques to make impressions for crowns and fixed partial dentures.

Various techniques have been highlighted in the literature regarding impression-taking for maxillectomy defects [16,17]. This includes the use of conventional impression techniques using materials, such as irreversible hydrocolloids (alginate), additional silicone polyvinyl siloxane (PVS), and polyether (PE). These conventional approaches are technique-sensitive and, depending on the size and extent of the defect, the clinician would usually seal off the defect with the use of a separating material, such as gauze to prevent any impression material from being dislodged. Alternative impression techniques have been introduced by the use of a two-piece special tray, individual customized special trays, and Computer-Aided Design and Computer-Aided Manufacturing (CAD/CAM) which can aid in impression taking even with the use of special attachments [18].

There is a lack of robust guidelines regarding impression materials and techniques for recording different types of maxillary defects. The aim of this study was to develop and optimize conventional and 3D-printed laboratory models of maxillary defects and to compare conventional and digital impression techniques using these models. The null hypothesis was that there is no difference between conventional and digital impression techniques for recording and replicating maxillectomy defects.

## 2. Materials and Methods

### 2.1. Defect Model Fabrication

**Figure 2:**
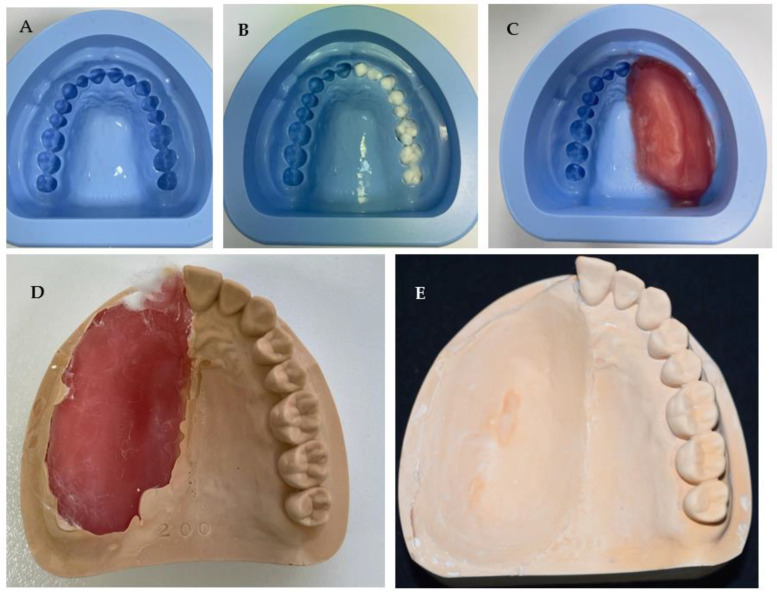
Laboratory steps of fabricating stone model of unilateral maxillary defect showing (**A**) silicone mould, (**B**) blocking the unwanted teeth for defect fabrication, (**C**) defect simulation with wax, (**D**) cast removed from mould after setting, and (**E**) completed model after trimming the unilateral defect.

In this in vitro study, a silicone rubber full-arch maxillary dental mould was used as shown in Figure 2A. Based on the Aramany classification, the maxillectomy defects were mimicked by blocking the unwanted teeth with cotton wool pellets (Figure 2B) and placing hard dental wax (Cavex Holland B.V. of Haarlem, The Netherlands) to block out the area of the defect (Figure 2C). Once the desired defect had been moulded, it was poured into type III stone and trimmed (Figure 2D), and the final stone model was produced after eliminating wax from inside the defect (Figure 2E).

A similar technique was used to fabricate six different types of maxillary defect models in stone, including midline defect, unilateral defect, central palate defect, bilateral anteroposterior defect, posterior defect, and anterior defect as demonstrated previously in Figure 1 in the introduction section.

**Figure 3:**
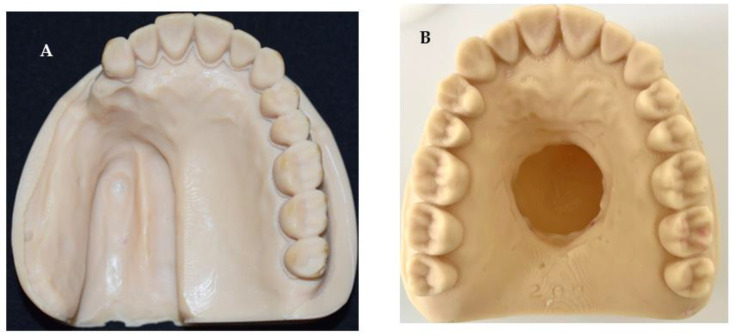
Images of 3D-printed models of maxillary defects showing (**A**) unilateral posterior defect and (**B**) a central palate defect model.

The stone models were then scanned using a desktop scanner (iTero Element scanner, Align Technology, San Jose, CA, USA) and plastic 3D-printed models were constructed using a 3D printer (NextDent 5100, 3D Systems, NextDent B.V., Soesterberg, The Netherlands) (Figure 3).

**Figure 4:**
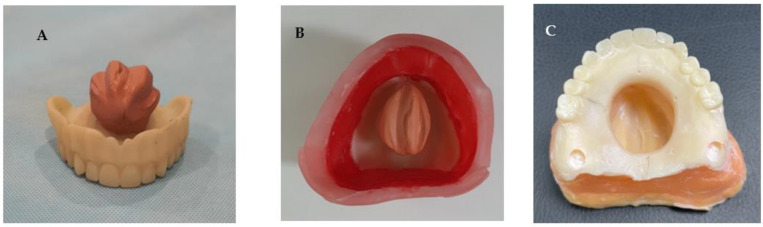
Laboratory steps of fabricating acrylic maxillary central defect model showing (**A**) the copied denture and the simulation of the central defect using silicon putty, (**B**) Boxing of the model to aid casting, and (**C**) acrylic maxillary central defect master model.

An acrylic central palatal defect model was also fabricated using a modified denture copy technique as described below:

A maxillary complete denture was copied using the flask technique, and silicon putty was shaped and placed in the palate to resemble the central defect (Figure 4A). Before processing, boxing of the master model was conducted to help in the pouring as shown in (Figure 4B). The model was then poured with cold cure acrylic resin material (GC Corporation, Tokyo, Japan) (Figure 4C). 

This master model was used to compare conventional silicon impressions with digital intra-oral scanning in terms of dimensional accuracy and total time taken to record the defect and produce a laboratory analogue. Four reference points were placed on the master model: anterior to the central defect, posterior aspect of the defect, and two in the lateral positions. The points in the defect were used to aid in measuring the dimensional accuracy between both techniques.

### 2.2. Sample Fabrication for Comparison Experiments

#### 2.2.1. Conventional Impression Technique 

**Figure 5:**
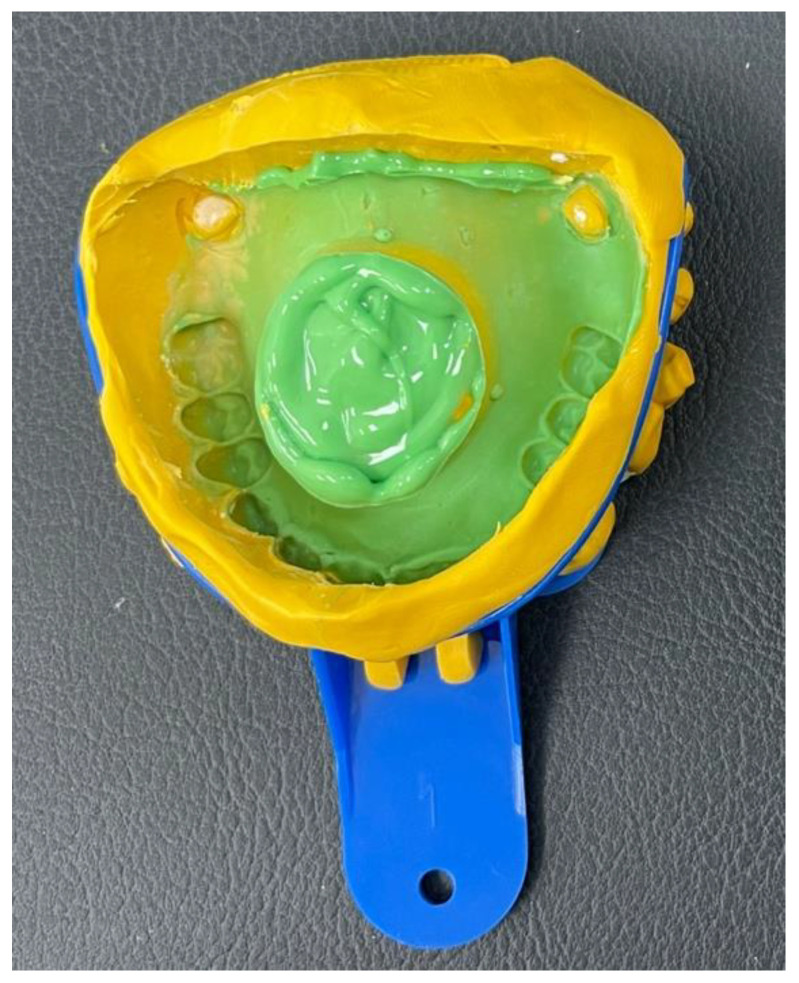
Conventional impressions were taken with heavy and light PVS impression materials using a two-step technique.

Poly vinyl siloxane (PVS) (3M ESPE, Saint Paul, MN, USA) heavy and light body impression materials with a two-step technique was used to duplicate the master models five times (*N* = 5) (Figure 5).

Different timings were recorded, such as mixing time, setting time, wash time, boxing time, and casting time. The purpose of recording these times was to calculate the total time used to fabricate such a model and use it in comparison with the digital technique. After boxing each impression, a stone model was poured to produce the duplicate casts. 

#### 2.2.2. Digital Technique 

**Figure 6:**
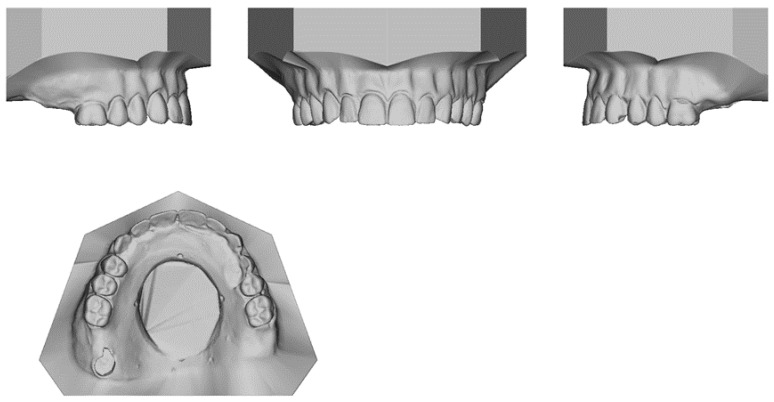
STL images of the digital scans showing the reference points that used in this experiment.

**Figure 7:**
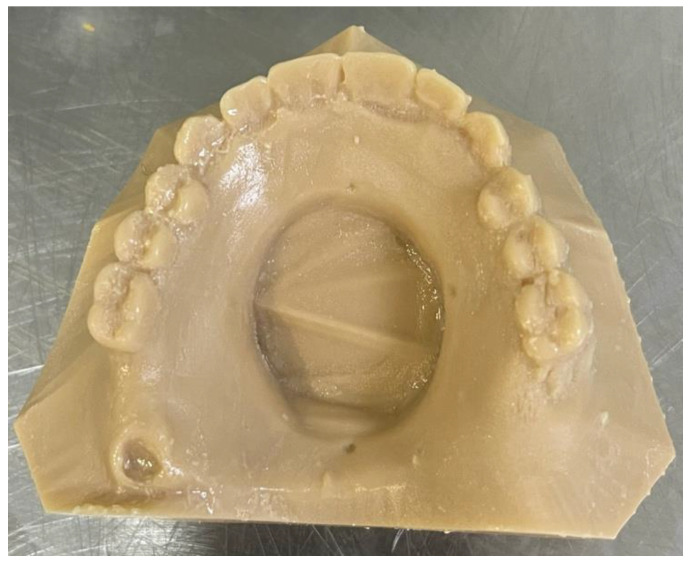
Image of the 3D-printed cast showing the central palatal defect.

In this part, the acrylic master model was scanned with an intra-oral scanner (iTero Element scanner, Align Technology, San Jose, CA, USA) five times. Five different STL files were generated (Figure 6). The STL files were sent to the 3D printer software (NextDent 5100; 3D Systems, NextDent B.V., Soesterberg, The Netherlands) each STL file was printed to produce resin models as shown in Figure 7. 

After full printing, the cast was placed in alcohol solvent to remove the uncured resin and then was placed in light curing machine to produce the final duplicated model. 

Digital scanning time, actual printing time, solvent washing time, and light curing time were measured to calculate the total timing taken to fabricate each model.

### 2.3. Measurement Collection

**Figure 8:**
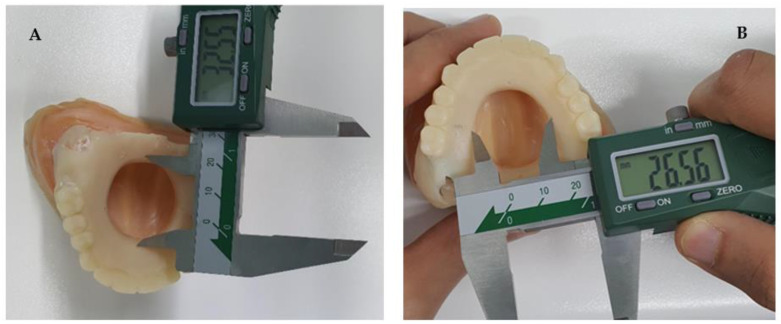
Digital micro caliper used to measure (**A**) the AP distance and (**B**) the lateral distance.

After fabricating 5 conventional casts and 5 digitally printed casts that were duplicates of the master models, a digital micro caliper (Mitutoyo, Kanagawa, Japan) was used to record the distance between the reference points. Two measurements were recorded: anterior–posterior measurement (AP) (Figure 8A) and lateral measurement (Figure 8B). 

### 2.4. Statistical Analysis 

In this study, the analysis of the fabricated casts evaluated the following parameters: Dimensional accuracy using reference points in both AP measure and Lateral Measure and the duration of impression taking and production of maxillectomy defect models using conventional impression techniques and digitized workflow.

Data were entered into the computer using IBM-SPSS for Windows version 28.0.0.0 (190) IBM (SPSS Inc., Chicago, IL, USA). The measurements of AP, lateral, and total time were described by measures of tendency and measure of dispersion. Shapiro–Wilk–Smirnov was used to test the normality of continuous variables. One independent sample t-test was used to compare means between standard, conventional, and digital methods. Pair t-test was used to compare pairwise between conventional and digital methods for total time, AP, and lateral measurements. Coefficient of variation was calculated to show the extent of variability in relation to the mean of the population and to measure the accuracy of digital and conventional techniques. A *p*-value of less than 0.05 was considered significant in all statistical analyses.

## 3. Results

The descriptive statistics are shown in Table 1, including the mean and standard deviation for each group and the measurement points. For the conventional techniques, the mean of the time per second is 3688.6 (37.85) seconds, while it is 3707 (139.48) seconds among digital techniques. For AP measurement, the average among the conventional techniques is 32.03 (0.24) mm, while it is 32.83 (0.11) mm for digital technique. The lateral measure has an average 25.85 (0.09) mm among conventional and 26.58 (0.11) mm among digital techniques. 

Analysis of the accuracy of the techniques was performed using the coefficients of variation. The descriptive statistics indicated that G2, G5, and G6 had higher coefficients of variation.

Table 2 shows the mean and standard deviation per case with conventional and digital techniques. Analysis of the accuracy of the techniques is performed using the coefficients of variation. The descriptive statistics indicated that cases C4 and C5 among digital techniques have the smallest coefficients of variation that indicates the extent of variability in relation to the mean of the population. The accuracy with digital techniques is better tendency wise. 

Table 3 shows the comparison of AP measure and lateral measure between standard and conventional from one side and between digital and standard methods from the other side. The result revealed that for the AP measure, the conventional method yielded a lower distance than the standard and the *p*-value was 0.008. A significant difference was also found between the digital technique and the standard with a *p*-value of 0.005. 

For the lateral measure, conventional techniques yielded a significantly less measure on average compared with the standard with the *p*-value of <0.001. No significant difference was detected between digital techniques and the standard for the lateral measurements as the *p*-value was 0.435. 

Table 4 summarizes the statistical comparison of AP, total time, and lateral measurements between conventional and digital methods.

## 4. Discussion

Many dentists are not trained to the standards of experienced prosthodontists and lack knowledge on treating patients with maxillectomy defects even though they are widely used in rehabilitating large maxillary defects. A study carried out on graduating third-year dental students highlighted that only 36% of students had heard the term ‘obturator’ in their final year, and 75% had not come across any patient requiring one [19]. This may be due to the scarcity of these defects. Studies have shown that malignant tumours of the maxilla are relatively uncommon, accounting for less than 6% of all head and neck tumours, with squamous cell carcinoma accounting for more than 90% [20]. Moreover, as graduating general dental practitioners, it is crucial to educate students on rehabilitating maxillectomy defects. Many clinicians lack knowledge and clinical guidance regarding an obturator because there are no standard teaching models designed to teach students at the pre-clinical stage. Furthermore, there is a lack of patients who require obturators as evidenced by Rogers et al. in 2022, who reported that even large-scale hospitals in the UK only manage less than ten of these cases per year on average [21]. Even if students and general dental practitioners have not come across obturators or patients with maxillectomy defects, it would be beneficial to train them on models that they might need to employ if a patient under their care does require such a prosthesis.

Therefore, there is a need to fabricate standardized laboratory models of different types of maxillectomy defects which can be used in the future for pre-clinical teaching of dentists and prosthodontics trainees.

The trend of using digital technology has spread to most dental clinics, hospitals, and laboratories. Digital technology has proven to be a viable option in order to take impressions or fabricate prostheses. However, research must be conducted to determine its true effectiveness using an evidence-based methodology. This in vitro study is considered to be the first study to determine the accuracy of conventional versus digital techniques in recording a central maxillary defect using standardised laboratory models. 

In this study, defects based on Aramany’s classification were constructed since they would be appropriate for teaching and training students as this classification system provides a wide variety of horizontal intra-oral maxillary defect configurations compared to Brown’s classification. Additionally, the four categories (a, b, c, and d) of Brown’s horizontal classification of maxillary defects are already covered by the current selection of maxillary defect models in this study. Replicating Brown’s vertical classification of mid-face abnormalities would be outside the scope of this manuscript. In 2012, a systematic review was conducted with the goal of evaluating classification systems in the literature to identify a universal description of maxillectomy and midfacial defects. Based on criteria encompassing both surgical and prosthodontic needs, no classification program has correctly described the maxillectomy defects. This highlights the need for more teaching about these defects and a universal system that can be used by both surgeons and prosthodontists to facilitate treatment planning [22].

In this present study, 10 models were fabricated using both conventional and digital methods. There was a significant difference in the impression time between both methodologies. It was recorded that a mean of 10 min 41 s needed in the laboratory for conventional impression taking, while in the digital methods, a mean digital scanning time was 3 min and 23 s. These results are consistent with the findings of Lee et al. who stated that the time of the digital impression method was shorter than the conventional impression method [23]. However, training must be provided to the dentist in order to achieve optimum outcomes. Providing training before taking digital impression will result in shorter duration of digital impression and, therefore, better time efficacy in comparison with the conventional methods [24].

Time comparison revealed that both techniques required similar time in total to fabricate a duplicate central defect model with a mean time of 1 h 1 m 26 s for the conventional technique and 1 h 1 m 47 s for the digital technique. This finding has not been previously reported and indicates that in some cases, digital technology and 3D printing may be as time consuming as conventional techniques. 

In this study, another comparison was used to determine the accuracy of both techniques by measuring the AP distance and lateral distance in the defect models using a digital calibre. The results indicated that the data collected was not significantly different in the AP measure, while it was significantly different in the lateral measure. The measurement taken in the lateral ends favoured the digitally produced cast over the conventionally made model. However, these results slightly differ from a systematic review that compared the accuracy of conventional versus digital techniques and included six studies to determine which technique was more accurate [25]. The study concluded that the conventional technique had higher precision and accuracy in comparison with the digital, hence rejecting the null hypothesis. Yet the study also supported that additional research should be conducted to produce more conclusive data and assess the accuracy in different clinical settings [25].

Another factor that may play a role in the accuracy of the digital technique was the intra-oral scanner. Scanning the palate appeared to be more technique sensitive than a normal full-arch dentate model without a palatal defect as there was lack of adequate landmarks in the defect area for the intra-oral scanner to use to easily orientate the scan. Repeated scanning movement toward the tissue was necessary to capture the defect and its surroundings. Given the size of the intra-oral scanner and the size of the defect, it was not possible to fully insert the scanner head into the defect and a fair distance was needed. These findings indicate the challenges that clinicians may face during scanning small to medium-size palatal defects where the head of the intra-oral scanner is larger than the size of the defect and the use of small-size scanner tips may be particularly beneficial in these cases. There are some studies in the literature investigating the scanning of the edentulous areas of the mouth and denture-bearing areas. A systematic review by Rasaie et al. was conducted to assess the accuracy of intra-oral scanners for recording the denture-bearing areas in both clinical and laboratory settings, which included 18 studies eight of which were clinical [26]. The study found the accuracy results were different when using different intraoral scanners especially when trying to capture denture-supporting areas and peripheral mobile tissues. Additionally, the study indicated that there were factors that could improve and aid in the ability to capture better impressions, such as using some artificial markers, scanner head size, scanning strategy, and the operator’s experience. It was also concluded that they were not capable of accurately registering the mobile tissues. This is particularly important when recording a maxillary defect to fabricate an obturator prosthesis as intra-oral scanning of palatal defects with mobile peripheral tissues may results in a suboptimal impression and subsequently making an obturator prosthesis with a compromised seal.

Nonetheless, digital techniques have been used recently to fabricate obturators from Polyetheretherketone (PEEK). Costa Santiago reported that the use of PEEK significantly facilitated the fabrication of the antral section of the palatal prosthesis, resulting in a significantly lighter obturator prosthesis, resulting in satisfactory patient comfort. However, in this clinical report, obtaining an impression of the orosinusal defect was challenging and the patient’s obturator prosthesis was used and duplicated from a silicone mould. The antral portion of the artificial palate was machined using a prefabricated PEEK disk and a duplicate of the patient’s obturator prosthesis as a model. This technique combined conventional and digital methods and solely did not rely on a digital method in scanning and recording the defect due to its complexity. A 6-month review revealed a satisfactory prosthesis and esthetics with no leaking or blockage [27].

Ye et al. described a fully digital workflow for the design and manufacture of maxillectomy defect prostheses in 2021. They scanned the maxillofacial region with spiral computed tomography (CT). PEEK was chosen for its material properties, which include strength, biocompatibility, and an elastic modulus comparable to cortical bone, making it suitable for fabricating a low-weight obturator as well as an acceptable clasp retention material [28]. However, PEEK’s esthetics was lacking. This can be enhanced by using resin or designing a multicoloured obturator prosthesis in 3D [29].

An additional drawback of this technique was that a reverse engineering software program was required in addition to the dental CAD software program which increased the learning time for a new creator and was technique sensitive. Furthermore, in many clinical circumstances, the clinician uses a combination of traditional techniques and digital technology to create an accurate prosthetic restoration, reducing treatment time, patient discomfort, and anxiety when constructing a definitive obturator prosthesis. Other patient factors, such as pain and trismus, which are prevalent in these patients, will favour digital technology over traditional technology due to its comfort [30].

These findings indicate that although digital technology has many advantages in prosthodontics, clinicians must be cautious and not always consider that digital technique is superior to conventional methods without appropriate pre-operative assessment of the intra-oral tissues and the defect anatomy. 

## 5. Conclusions

Within the limitation of this study, the stone and 3D printed plastic models and the acrylic replicate of the maxillary defects that were successfully produced in this study appeared to be suitable laboratory models to represent different configurations of maxillary defects which can be used to study the differences between conventional and digital workflow in prosthetic treatment procedures. Digital techniques to record and fabricate a central palatal defect model may produce different results than the conventional technique in terms of defect size measurements. Although the time taken to record the arch and the defect using an intra-oral scanner is significantly less compared with the traditional impression method, the total time taken to fabricate a maxillary central defect model may not be significantly different. 

## Figures and Tables

**Table 1:** Descriptive of measurements.

	Conventional	Digital
Items	Mean (SD)	Mean (SD)
Time per second	3688.6 (37.85)	3707 (139.48)
AP measure (mm)	32.03 (0.24)	32.83(0.11)
Lateral measure (mm)	25.85 (0.09)	26.58 (0.11)

**Table 2:** The mean, standard deviation (SD) of different time, and coefficient of variation (CV) per case.

		Conventional		Digital	
Case	N	Mean (SD)	CV	Mean (SD)	CV
C1	5	741.8 (1102.24)	1.49	1119.4 (934.55)	1.04
C2	5	738.4 (1103.19)	1.49	1138.6 (982.13)	1.08
C3	5	747 (1099.89)	1.47	1160 (955.09)	1.00
C4	5	727 (1108.54)	1.52	1134 (925.25)	0.99
C5	5	734.4 (1104.35)	1.50	1089.2 (856.70)	0.94

**Table 3:** Comparisons measurements between conventional and digital methods with the standard measurements.

		Conventional		Digital	
Measurement	Standard	Mean (SD)	*p*-Value	Mean (SD)	*p*-Value
AP measurement	32.55	32.03(0.24)	0.008	32.83(0.11)	0.005
Lateral measurement	26.54	25.85(0.09)	<0.001	26.58 (0.11)	0.435

**Table 4:** Comparison of AP, total time, and lateral measurements between conventional and digital method.

	Means	SD	95% CI of Difference		*p*-Value
Pairs			Upper	Lower	
AP-conventional-AP-digital	−0.80	0.22	−0.52	−1.08	0.001
Lateral-conventional-Lateral-digital	−0.73	0.09	−0.62	−0.84	<0.001
Total time conventional-Total time digital	−18.60	127.54	139.76	−176.96	0.761
